# Improving Index-Based Drought Insurance in Varying Topography: Evaluating Basis Risk Based on Perceptions of Nicaraguan Hillside Farmers

**DOI:** 10.1371/journal.pone.0051412

**Published:** 2012-12-11

**Authors:** André Kost, Peter Läderach, Myles Fisher, Simon Cook, Lorena Gómez

**Affiliations:** 1 University of Bonn, Dept. of Geography, Bonn, Germany; 2 International Center for Tropical Agriculture, Managua, Nicaragua; 3 International Center for Tropical Agriculture, Cali, Colombia; 4 International Water Management Institute, Sunil Mawatha, Pelawatte, Battaramulla, Sri Lanka; New York State Museum, United States of America

## Abstract

This paper discusses a methodology to model precipitation indices and premium prices for index-based drought insurance for smallholders. Spatial basis risk, which is borne by the insured, is a problem, especially in variable topography. Also, site-specific drought risk needs to be estimated accurately in order to offer effective insurance cover and ensure financial sustainability of the insurance scheme. We explore farmers' perceptions on drought and spatial climate variability and draw conclusions concerning basis risk with regards to the proposed methodology. There are technically many options to represent natural heterogeneity in index insurance contracts while serving the customer adequately and keeping transaction costs low.

## Introduction

### 1. Agricultural Risk Management and Index Insurance

Index-based insurance for agriculture [1], [2], [3] was developed in industrialized countries to avoid adverse selection and moral hazard in traditional agricultural insurance [4], [5], [6]. The key is the index, which is commonly based on measured rainfall [7], [3], [5] as a proxy for yield shortfalls due to adverse weather. The index-insurance contract is not about crop loss, but a predefined range of values of the index. In the case of a precipitation index, insurance cover is based on the rainfall measured by a gauge at a predefined site.

Index insurance lowers transaction costs because farm inspections are unnecessary [8], and because the index is used to set the premiums and assess any indemnity. Low-cost index insurance is a possibility for smallholder farmers in developing countries where insurance companies are few and have limited capacity, and where it is difficult to access rural areas because of poor infrastructure.

Although farmers are accustomed to manage risk, poor smallholders invariably use strategies that minimize it, typically by investing as little as possible in their subsistence crops. Index-based insurance could be a supplementary strategy [9] to allow them to reduce risk [10] and give them access to credit by hedging their investment in crop inputs. Index insurance could also partially substitute for emergency aid when there are catastrophic harvest failures [10], [11].

There is a 14-year history of testing index insurance in Nicaragua for commercial crops of rice, peanuts, maize, soy, and sorghum in the western lowlands using a statistical approach [5], [12]. In 2009, the total sum insured was about US$2 million, but the scheme was not for poor smallholders. It was carried out by the public Nicaraguan insurer INISER and the World Bank [3]. In the same year, the international insurer Lafise opened first the national market for agricultural insurance within an initiative of the Interamerican Federation of Insurance Companies (FIDES), insuring more than 700 ha of irrigated rice and peanuts. In 2010, coverage was expanded to maize, beans, and sorghum. Insurance was supposed to be sold to producers of any type via cooperatives and local financial institutions. By this means, it was expected to reach around 5000 producers, mainly in the central and northern parts of Nicaragua [13], [14], [15].

However, a statistical approach, which Lafise and INISER currently use, may not be sufficiently precise to determine a farmer’s risk of crop loss [16], [17]. This is even truer where the topography and microclimates are heterogeneous as it is the case for north-central mountains of Nicaragua. In this study, we explore a more precise method using a weather generator and crop physiological models to determine crop risk, which is applicable to any other place in the tropics and to a large number of crops. Specifically we explore farmers' perceptions on drought and spatial climate variability and draw conclusions concerning basis risk with regards to the proposed methodology.

### 2. Study Context

Drybean is an important subsistence and cash crop of smallholder farmers throughout Central America. Surveys of smallholders in western Matagalpa department in central Nicaragua showed that they knew little about insurance of any sort [18,unpublished data]. They quickly understood how index-based crop insurance works and expressed interest in it to protect them against yield loss of rainfed (not irrigated) drybean crops. Although farmers were hypothetically willing to buy insurance cover, this does not guarantee that they would buy it were it to be offered. It is noteworthy that an innovation introduced from the exterior may not be adopted, often because it does not work well at a particular locality [19].

The major problem of index-based insurance is that it exposes the insured to *basis risk* [16], [17] in which the damage caused by the insured event is worse for the insured than at the point where the index is measured [20]. The insurer is covered in that the premiums are calculated on an actuarial assessment of the risk of the insured event occurring at the same point where the indemnity is assessed, the long-term meteorological station. It is the insured who bears the basis risk [16], [17], which is an aspect of index-based insurance that has been neglected. If an insurance instrument has a high basis risk, it cannot fulfil the clients’ expectations, and will therefore not sell [21].

The sources of basis risk in agricultural index-insurance based on precipitation are temporal risk, crop-specific risk, and spatial risk [22]. To reduce basis risk, index developers must consider (1) the temporally-varying water needs of (2) specific varieties and (3) the spatial variation of water availability due to natural heterogeneity.

The temporally-varying water needs of specific varieties ((1) and (2) above) are captured by the Decision Support System for Agrotechnology Transfer (DSSAT) simulation software [23]. DSSAT combines current knowledge of agro-ecological systems, crop agronomy, and plant physiology. It can be used to construct indices more consistently than statistical approaches [16], [17]. Reducing spatial basis risk is harder, especially where there are only a few scattered meteorological stations with poor historical data, which is often the case in developing countries. It is even more difficult in heterogeneous topography. The current best solution is to extract monthly data from the 1-km-resolution climate data in the WorldClim database [24] and use them as input to the MarkSim weather generator [25], [26], to produce daily weather data. It costs more to design site-specific indices, which increases premiums, but the index must be sufficiently site-specific that the buyer considers the basis risk to be negligible [27].

In this study we investigated basis risk using Díaz Nieto et al.’s methodology for index-based drought insurance for drybean [16], [17], [18] as a starting point. We explored farmers’ perceptions and preferences on crop risk and insurance to identify what aspects of the methodology require further development. By this means, we sought to “bridge the two cultures of risk analysis” [28]: to integrate traditional numerical risk analysis with the layman’s perception of risk to implement a sustainable process of risk management [29].

### 3. Modeling Precipitation Indices for Nicaraguan Drought Insurance

**Figure 1:**
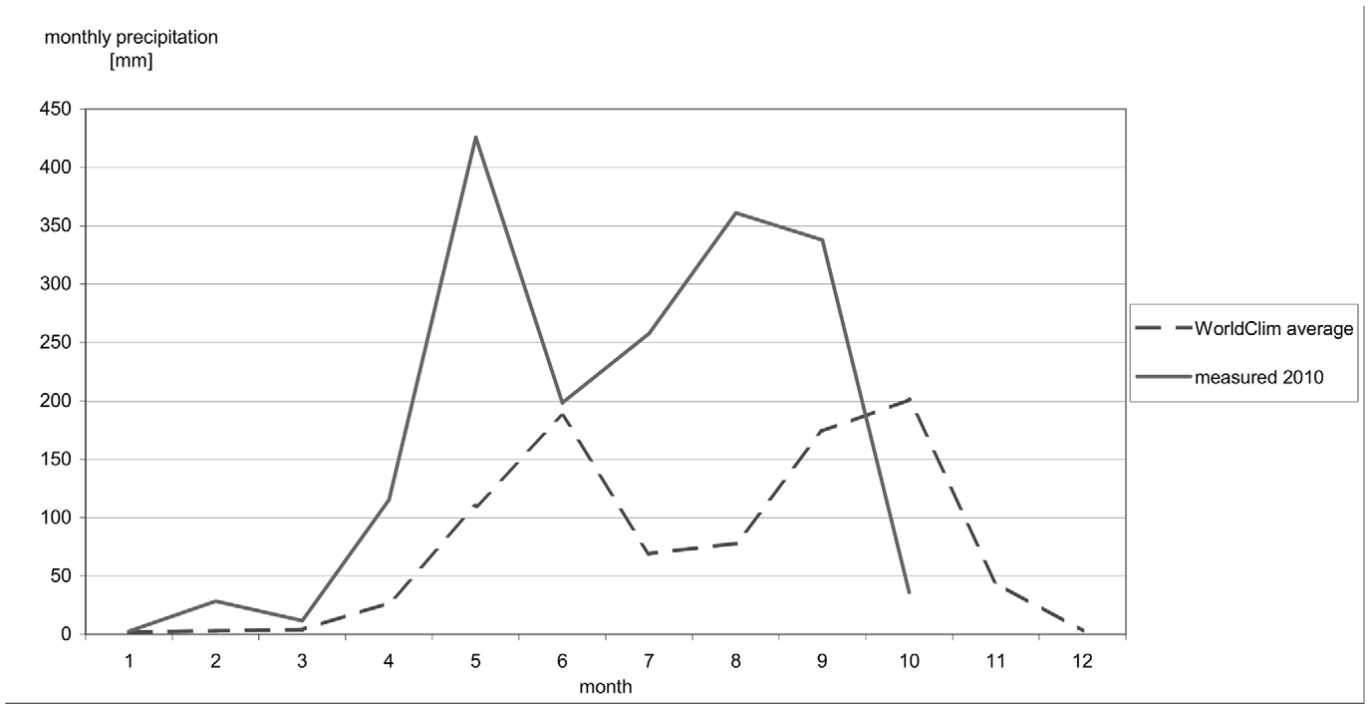
Precipitation January-October 2010 at weather station San Isidro (458 m). (Source: WorldClim database and [**51**] **).**

The north-central mountains of Nicaragua have an east-west precipitation gradient, with the highest annual rainfall (2000 mm) on the eastern slopes and the lowest precipitation in the valleys. A sequence of intramountain plains extends from Ciudad Dario northwest towards central Honduras forming the *franja seca* (dry fringe) with annual rainfall less than 800 mm [30]. The *franja seca* crosses the study area in the municipality of San Isidro. Rainfall is distributed bimodally with peaks in June and September/October and a relative dry spell July – August (*canícula*) [31]. There is considerable variation in precipitation in any one year (Figure 1). For example, the start of the rainy season in May and its end in November is influenced by the current status of the El Niño-Southern oscillation (ENSO). El Niño corresponds with years of extensive drought [32], while La Niña corresponds with above-average rainfall [33], such as in 2010 when a strong La Niña gave damaging high rainfall totals and in which there was no *canícula*.

Tropical rainfall mainly depends on convectional cells 100–300 km^2^ in extent. These cells remain stationary for a few days and give high-intensity rainfall events that only last for a few hours. In general, the convection cells occur randomly but they are also influenced by topography [34]. In view of this, a particular raingauge only represents a relatively small area. Indeed, two places only a few kilometers apart can have completely different rainfall patterns over periods as long as one month [34]. What, then, are the consequences for spatial basis risk of any index insurance scheme?

In the sub-humid tropics the performance of rainfed agriculture in any one year is determined primarily by the characteristics of the rainy season and temperature, which is the main driver of evapotranspiration. Temperature variations with altitude can also be important in mountainous regions. The impact of rainfall on crop production depends on daily soil water balance [34], which requires daily meteorological data, and highlights the importance of modeling soil water to obtain a valid insurance index.

The challenge in the design of an index in a heterogeneous landscape is to balance cost and benefit, that is, whether to represent the variation with more precision, and cost, to reduce the basis risk. On the other hand, even a quite elaborate index can have high basis risk if the spatial scale is not appropriate [27], [35]. The DSSAT simulation model provides a cost-effective method to produce indices that take account of variable natural conditions at an appropriate scale to reduce basis risk in a transparent manner.

Díaz Nieto et al. [16], [17], [18] used the weather generator MarkSim and the DSSAT drybean submodel to construct precipitation indices for drought in Central America based on ten-day rainfall weights (Table 1; see also [16]). They extracted the probabilities of a given rainfall deficit from 99 years’ generated daily weather data at 18-km resolution.

**Table 1:** Weightings of ten-day precipitation in the DSSAT averaged insurance index for drybean.

	Day 1–10	Day 11–20	Day 21–30	Day 31–40	Day 41–50	Day 51–60	Day 61–70	Day 71–80	Day 81–90
**crop stage**	**planting/seedling**	**Seedling** **/flowering**	**flowering**	**Flowering** **/grain fill**	**grain fill**	**grain fill/maturity**	**maturity**	**maturity**	**maturity**
**index values [mm]**	10	10	25	40	40	40	30	10	0
**index weighting (rounded)**	0.05	0.05	0.12	0.2	0.2	0.2	0.15	0.05	0

(Source: adapted from [17]).

We used the same method, but with a spatial resolution of 1 km by extracting climate data from WorldClim [24].

## Methods

### 1. Research Focus

Farmers are more likely to accept an index insurance product if it is designed with their participation. Although this costs more to set up, it has the advantage of exposing farmers to the new concept prior to launching the product [7], [36], [37]. We interviewed smallholder households, whose main crop was rainfed drybean and who are likely to be potential buyers of index insurance, with the purpose to evaluate the basis risk inherent in index insurance as described above. We focused on four areas:

1. Exploration of farmers’ notions of drought.
2. Investigation of factors, apart from precipitation, that farmers perceive to influence yield. How do farmers perceive the importance of weather risk/drought risk compared with other risks?
3. Assessment of the plausibility of the averaged precipitation index for drybean crops developed using the Díaz Nieto et al. methodology [16], [17], [18]. The guiding question was how important do farmers judge the right rainfall distribution to be to obtain good drybean yields?
4. Investigation of farmers’ perceptions of spatial climate variation. What conclusions can be drawn for the assessment of spatial basis risk of an index-based insurance product?

### 2. Perception of Environmental Risk

**Figure 2:**
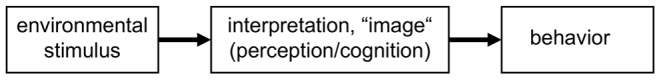
Behavioristic stimulus-response-model with perception component. (Source: adapted from [**36**]).

In theory, perception and cognition are variables in a behavioural stimulus-response-for explaining human behaviour (Figure 2). There is a (rather conceptual) distinction between how an individual sensorially *perceives* the environment (i.e. perception) and how this information is further processed mentally, which is *cognition* [38]. As an individual’s *environment* we define “the total milieu in which man lives, an environment with both physical and sociocultural attributes” [39]. Consequently, “the key intervening variables in man – environment transactions are perception and cognition – the internal mental processes by which individuals sense, perceive, interpret, and make decisions about their environment” [38].

In this study we deal with a broader understanding of *perception* “in the sense of how things are remembered or recalled by people - as with respect to 'perception' of resources or hazards” [39]. In this sense, perception denotes the mental representation one has of one’s environment. It is not therefore intended to explain a certain behavior through perception, but rather to investigate a subject’s mental images.

We are concerned here with farmers’ perceptions of site-specific risks in rainfed drybean. “Risk perception, in general, denotes the processing of physical signals and/or information about potentially harmful events … and the formation of a judgement about seriousness, likelihood and acceptability of the respective event” [29]. In this case we considered climate events that reduce yields.

We could not capture farmers’ perceptions of basis risk directly because we judged that they could not understand the concept within the time limitation of a single interview. Thus, farmers could not evaluate the insurance model directly. Instead, we sought to employ farmers’ perceptions of the environment as a proxy for their perception of basis risk by using qualitative methods for analysis of verbal data [40], [41].

### 3. Research Design

The climate and topography in western Matagalpa vary considerably within short distances, making it suitable to test whether WorldClim and MarkSim represent the spatial variation of rainfall patterns and realistically model the probability of drought. We also evaluated the suitability of the averaged precipitation indices that we calculated.

**Figure 3:**
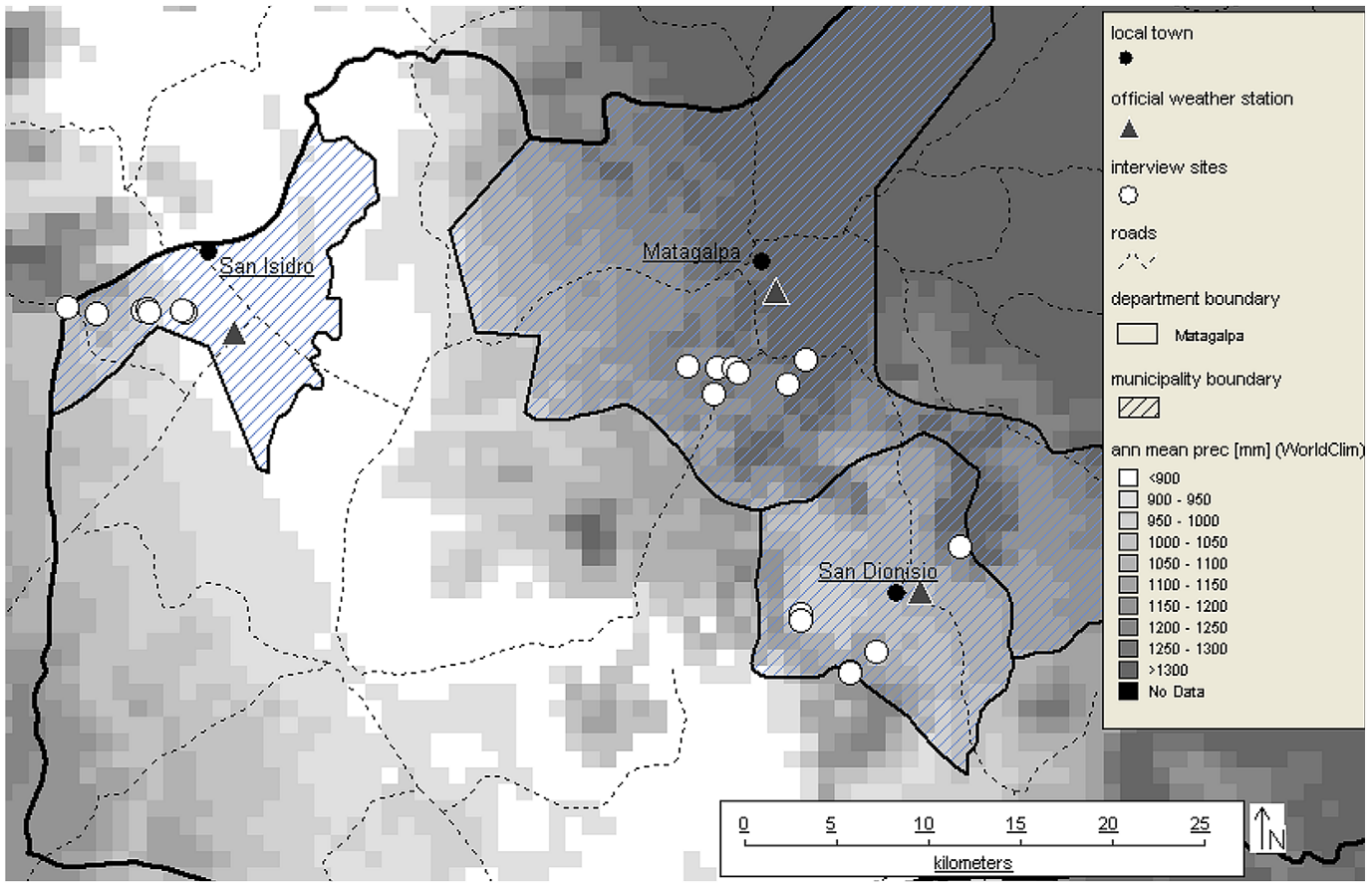
Mean annual precipitation in the municipalities of Matagalpa, San Isidro, and San Dionisio. (Source: WorldClim database, prepared with DIVA-GIS).

The municipalities of Matagalpa, San Isidro, and San Dionisio, all within 30 km, encompass the climatic variability of the region. Within them we chose sites having contrasting climates to sample a wide range of climate risk and maximize variance [40]. Moreover, each site has an official weather station so that they could hypothetically serve as reference gauges if an index insurance scheme based on precipitation were to be established (Figure 3).

We held six meetings with drybean farmers in each municipality, with no more than two farmers from any one neighbourhood or community. The meetings were organised as “problem-centred” interviews [42], which focus on a specific problem, in this case perceptions of the effect of weather on drybean yields. Problem-centred interviews need a certain degree of structuring but they also foster narrative flow, which helps in creating confidence and lessens the interview’s artificiality [42].

Data analysis and interpretation follow Mayring’s “qualitative content analysis” [41] and established typologies [40], both of which interpret verbal data by building systems of thematic categories. Categories, in turn, were derived deductively from research hypotheses, which we formed in advance or alternatively created *ad hoc*, inductively if necessary [40]. In the final step we linked categories to build a system of theorizing statements (typologies).

As an example, we defined a typology of households that captured their distinctive features: (1) the extent of a household’s resource scarcity, and (2) the household’s attitude towards its resource scarcity. This gives four types of households, which we named *pioneers* (young families, scarce resources, optimistic attitude), *established* (families with grown-up children, sufficient resources, modest or indifferent attitude), *precarious* (drought-prone, scarce resources, ironic or pessimistic attitude), and *go-aheads* (leadership tasks in community, sufficient and diversified resources, cooperative and innovative attitude).

## Results

We first present a short overview on household characteristics and farming practices for which we use data from our survey (cf. 1.). After this we elaborate the interpreted verbal data with reference to the points listed in chapter 1 of the methods section in four subsections (cf. 2.–5.).

### 1. Households’ Farming Systems and Economic Behaviour

The farmers grow drybean in two periods during the rainy season, the *primera* (May–August) and the *postrera* (September–November). Normally, the *primera* crop is harvested in the *canícula* (July–August) and the *postrera* is harvested in the first weeks of the dry season (November–December). Occasionally, farmers opportunistically grow a third *apante* crop, sown in November/December and harvested in February, but yields are low, the area sown is less than 0.7 ha.

Farmers normally sow drybean 20 May–10 June (*primera*), 1 September–5 October (*postrera*), and 10 November–15 December (*apante*) [43]. Individual farmers may use narrower ranges, especially in hotter locations for the *postrera*. Farmers typically wait for three to five consecutive rainy days to have sufficient soil water to sow the crop. Harvest is 60–90 days after sowing, with crop duration shorter in warmer climates (Table 2), which are often also drier. Duration of the *postrera* crop is 10 days shorter than the primera.

**Table 2:** Climate and bean cultivation characteristics at 18 interview sites.

Interview site		Climate (WorldClim)			Bean cultivation characteristics			
No.	Community (Municipality)	Temperature (average/year) [°C]	Precipitation (May-Nov) [mm]	Altitude [m] (SRTM90)	sowing in *primera*	sowing in *postrera*	bean cycle [days]	grown varieties
7	Santa Rosa de la Lima (San Isidro)	24.1	877	486	1.–10.5.	15.8.–10.9.	60	criollo
8	Santa Rosa de la Lima (San Isidro)	24.1	877	488	1.5.–10.6.	8.–20.9.	60	INTA rojo
13	Las Cuchillas (San Dionisio)	23.5	965	593	1.–25.6.	1.–20.9.	75	INTA Masatepe
12	El Bocón (San Isidro)	23.1	950	674	1.–31.5.	1.–15.9.	60	criollo
14	Las Cuchillas (San Dionisio)	23.1	988	641	28.5.–15.6.	28.9.–15.10.	75	INTA Masatepe
16	Ocote Arriba (San Dionisio)	23	1032	685	12.5.–15.6.	1.9.–5.10.	60	INTA Masatepe
1	Limixto (Matagalpa)	22.4	1280	918	15.5.	1.9.-begin Oct	75	criollo
17	Wibuse (San Dionisio)	22.2	1278	637	1.–31.5.	1.–20.9.	85	INTA rojo
18	Wibuse (San Dionisio)	22.2	1278	637	28.5–23.6.	15.9.–9.10.	75	INTA rojo
11	El Bocón (San Isidro)	22.1	972	680	10.5.–13.6.	1.–15.9.	75–85	criollo
15	Ocote Arriba (San Dionisio)	21.9	1142	786	1.5.- begin June	1.9.–15.10.	90	INTA rojo
3	Jucuapita (Matagalpa)	21.8	1255	828	May-June	15.9.-begin Oct	70	Estelí
							85	H
2	Jucuapa Centro (Matagalpa)	21.2	1203	889	1.5.–15.6.	1.–30.9.	85–90	H
9	Las Sidras (San Isidro)	21.1	1043	965	10.5.–30.6.	1.9.–5.10.	75	INTA Masatepe
6	El Ocotal (Matagalpa)	20.5	1229	973	10.5.–4.6.	14.9.–4.10.	80–90	INTA Palma
10	Las Sidras (San Isidro)	20.3	1179	1120	-	-	-	-
4	Nuestra Tierra (Matagalpa)	20	1390	1104	1.5.–20.6.	1.–20.9.	-	-
5	Santa Josefina (Matagalpa)	19.6	1352	1112	June	1.9.–15.10.	85	INTA Masatepe

(Source: WorldClim database, SRTM90 elevation data, own data).

Farmers’ households were 3–11 persons, usually the farmer couple and their non-adult children, and occasionally other relatives. All households cultivated the basic grains, drybean and maize, and in warmer climates also sorghum, often with some livestock. In cooler climates, households typically grew coffee as a cash crop. Many households grew some citrus, while the most diversified household had additional crops such as banana, sugar cane, cassava, and rice.

Holding size varied considerably, from landless households, which rent the land they cultivated, to a maximum of 55 ha, with larger holdings having more livestock. The area cropped with drybean is typically 1.5–2 ha in each season, with a maximum of 7 ha.

All households were risk averse. Economic priorities were always: (1) Securing household members' basic food security; (2) Settling outstanding liabilities; (3) Creating a reserve for the next crop; and (4) Providing for health care, clothing, and education. All households’ primary goal was to achieve food security with the basic grains and then to stabilise the household’s assets. This means that any cash income from the crop covers running expenses first and leaves neither scope nor incentive for investment.

Many farmers use short-term credit, either formally from an organization or informally from a friend or relative. Credit is not invested in productive assets, but is used only to cover emergencies, often only when there was no other way to finance the new sowing. These characteristics are typical of the poverty trap [44], [45] of persistent, long-term, economic stagnation. A household may accumulate some capital in the short- or mid-term, but unforeseeable ( =  risk as probability) and unavoidable ( =  risk as exposure) shocks counteract any attempt at economic expansion [45]. Economic growth is impeded by the existence of uncontrollable risks, which is how the farmers perceive them, whose potential damage exceeds a household's ability to cope.

### 2. Farmers’ Notions of Drought

Wilhite and Glantz [46] classify drought into meteorological, hydrological, agricultural and socioeconomic drought. Here, we fit farmers’ notions of drought to this scheme.

1. Meteorological drought: For the farmers, “drought” is marked by the two dry seasons in the course of a year. The first is the long dry season December–April (*verano*), the second is the short *canícula* dry period.
2. Hydrological drought: Farmers associated dried-up streams with a drought, concluding that there was no rain even in the uplands.
3. Socioeconomic drought: Streams that had run dry had serious consequences for everyday life since there were shortfalls in drinking water, which constrains all economic activities.
4. Agricultural drought: Drought just after sowing of the drybean crop gives poor germination, so that farmers delay sowing until there is sufficient soil water, because they cannot afford a failed sowing. In some localities with favorable soils, drybean crops can withstand dry periods for as much as one month after emergence. Drought at harvest is desirable to get good grain quality, while rain at harvest reduces both yield and quality. Nevertheless, most crops give some yield in high rainfall years, and coffee performs better. Farmers fear drought because it causes food insecurity and leaves no agricultural alternatives to produce income.

### 3. Perceptions of Yield-determining Factors

We investigated how farmers manage their drybean crops, hypothesizing that both agroecological processes and daily household routines play key roles, as well the households’ perception of weather risk. There are three systems:

- Households that grow drybean as a component of food security, typically a specific variety to satisfy their household’s culinary preferences of taste and cooking consistency;
- Households that only produce grain for sale, usually a specific variety that yields best or commands a market premium;
- Households that sell the surplus to their food needs. Some of these households cultivate two varieties, one for consumption with the preferred culinary characteristics, and a second for sale with better yields or higher price.

Farmers do not have information on the yield potentials of varieties suitable for marketing against which they can assess the performance of their crops. Nor do they have ready access to new varieties (see also [47]). They therefore usually refer to benchmark yields derived from their own experiences with one variety, which they have likely grown for decades. Only those households classified as “go-aheads” obtained benefit from the national extension service, which installed experimental plots of improved varieties in farmers’ fields. The experiments tested the varieties' local adaptation and provided objective yield benchmarks.

Farmers know that drybean responds to fertilizers of different qualities, pesticides, soil fertility, hill slope, and variety, all of which are under their control. They know that they can choose the sort and quantity of inputs, soil conservation measures, which lots to cultivate, and which variety to grow. In reality, however, both their limited perception of agroecological processes, especially yield responses, and socioeconomic necessities limit their ability to increase yields. Moreover, people will only seek to innovate to solve a problem if they recognize that indeed there is a problem.

Farmers agreed that the distribution of rainfall in the wet season was the main factor influencing yields, indicating that they accepted having no control over climatic risk, which they did not perceive as a problem needing resolution. Reliable weather forecasts can help farmers better estimate climate risk in the short term, for example to adjust sowing and harvesting dates [48]. Surveyed farmers used traditional dates even when weather forecasts were unfavourable, indicating that farmers believe that the national meteorological agency’s (INETER’s) forecasts were too approximate to be reliable. Moreover, INETER’s forecasts were often at odds with farmers' traditional forecasts. We could not verify that seasonal forecasts, broadcast on radio and television, influenced farmers’ decisions on crop management.

### 4. Perceptions of an Optimal Rainfall Distribution in Dry-bean Cultivation

**Figure 4:**
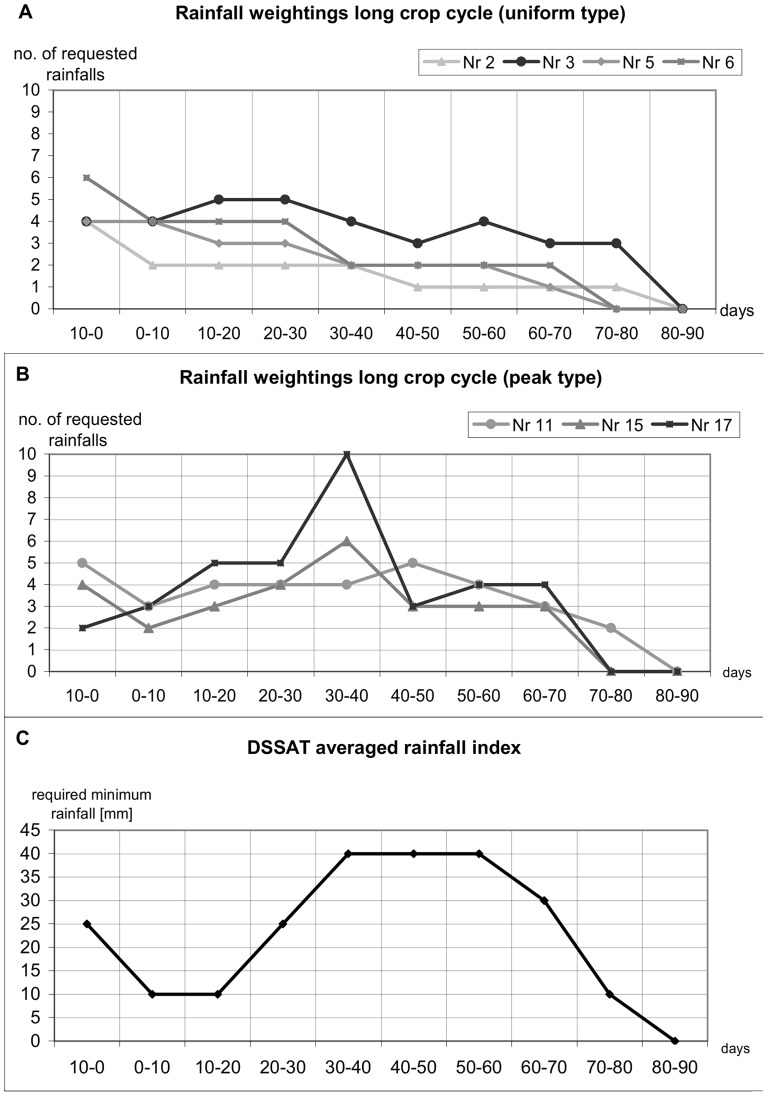
Optimal rainfall weightings in farmers’ graphs (A and B) and DSSAT averaged insurance index (C). (Source: own data, [**13**]).

We asked farmers to sketch optimal rainfall distribution (ten-day steps) that would give good yield and classified the graphed results. We also used them to cross-check the averaged rainfall index (Table 1, Figure 4) we developed [17].

Farmers agreed that good crop yields required evenly-distributed rainfall throughout the season, with a moderate (*normal*) total, with a few days of rain alternating with a few sunny days. A *normal* wet season gives *normal* yields of drybean. *Normal* yields varied between farmers as much as twofold in the same growing season in the same year (300–600 kg/ha) whereas maximum yields could be as much as1300 kg/ha. Yields in 2010 *primera* season ranged from total crop loss to about 1000 kg/ha.

Heavy rains rather than drought caused total crop failure, while all farmers indicated that there was always enough crop to cover the household's consumption needs. Nevertheless, in drought years yields were much less than normal.

The graphical comparison showed that drybean has differing crop durations across the research area (Table 2), with long duration (85–90 days, 7 farmers), medium duration (75 days, 5 farmers) and short duration (60 days, 4 farmers). Rainfall is most important in the 10 days before sowing, while dry conditions are required at harvest.

We distinguished two weighting patterns in the farmers’ graphs of crop water requirement (Figure 4). Panel B shows increasing requirement until the middle of the cycle and then declines (*peak* distribution), reflecting the physiological sensitivity of the bean crop to water deficits, which the farmers knew. Panel A shows almost constant demand (*uniform* distribution), which is consistent with crops grown on soils that have high available soil water. Farmers with this type of demand indicated that ample rainfall was important before sowing, but that a few evenly distributed rainfall events are sufficient thereafter. Yields in the drier areas were lower, requiring rainfall each 8–15 days.

### 5. Perceptions of Spatial Climate Variation

Climate information is available to farmers on spatial scales ranging from national, local, to their own on-farm perceptions. Farmers’ concept of climate is more than a location's meteorology, however. They typically responded that the climate is *normal* or *good*, meaning that they perceive it to be normal or good for the crops they grow, which emphasizes that they see agriculture and climate from a holistic perspective. Similarly, they often linked *climate* with *soil*, referring to soil properties, such as water holding capacity, when describing a farm's climate more precisely. This indicates farmers’ awareness of the linkage between rainfall and soil properties on crop performance [49], [50].

We delineated farmers’ spatial *frame of reference* [51] for climate. They commonly used four terms to describe climate at different locations: *helado*, *fresco*, *cálido*, and *caliente*, literally cold, fresh, warm, and hot. Farmers’ use of these terms includes the concept of both temperature and humidity, in that *caliente* means both hot and dry, and *helado* means not only cold but humid as well.

To many farmers a location’s climate is described in terms of its altitude relative the farmer’s reference point. If a location is higher (*más arriba*) than the farmer's own farm, the climate up there will be cooler (*más fresco*). Thus farmers in Matagalpa perceive spatial climate variation as a difference in altitude, and by comparing altitudinal levels, they construct small-scale homogenous climatic zones, e.g. *zona fresca* and *zona caliente*.

Farmers perceive soils to be better in higher locations in the landscape because they have more available soil water (ASW), calling them *tierras negras* caused by their higher organic matter content and higher ASW. At the other end of the scale are *tierras blancas*, which are sandier, have less organic matter, and lower ASW.

Farmers assume that rainfall originates near a mountain and spreads out from there to lower altitudes with decreased intensity and frequency. A farmer’s typical perception of spatially differing precipitation is relative (*more* or *less*) since they have little knowledge about rainfall gauging, but a perception that is linked to the perceived climate zones. They judge a weather station’s precipitation data to be representative of their farm if they perceive it to be situated within the same climatic zone, e.g. *zona fresca*. When extreme events occur, such as the high rainfall totals in 2010 or a drought, farmers perceive that all locations are affected similarly. In May, 2010, however, Matagalpa’s rainfall was 128 mm while San Isidro, 30 km distance, recorded 425 mm [52]. Further analysis of the meteorological records shows significant variability in monthly rainfall totals at nearby weather stations.

Farmers believe that hotter climates have more severe and more frequent droughts, which cause less loss because the hotter climates are less productive. In contrast, although less frequent, they believe that drought causes more damage within the more productive zones at higher altitudes with moderate climate.

## Discussion

We discuss the results presented above in relation to the insurance model. We first discuss how the two core components, the index model and the spatial model, correspond with farmers’ perceptions to highlight how farmers see basis risk as it applies to them (cf.1.–2.). We then draw on farmers’ perceptions to propose measures that lessen basis risk in the research area but which are also relevant elsewhere. Finally we relate farmers’ perceptions to model variables that need scrutiny and discuss some practical implications (cf. 3.).

### 1. Validity of the Insurance Index

Here we rely on the results of the farmers’ rainfall drawing exercise and we use *validity* in the context of *plausibility* of the insurance index from the farmers’ perspectives.

We assume farms classified as requiring *peak* rainfall distribution are based on some knowledge about physiological stages and related water needs of drybean. For these farmers, the averaged insurance index (Figure 4, Table 1) will probably be more plausible and hence more acceptable than for those whose farms were classified as requiring *uniform* rainfall distribution. The *uniform* distribution requirement may be interpreted in one of three ways:

1. Farmers did not know that water demands of drybean vary according to physiological stages, and they think that equally-distributed rainfalls lead to the best yields;
2. There is sufficient water supply at this site during the rainy season (it has low drought risk, and a cool microclimate); or
3. The site has soils with high ASW and a microclimate with low evapotranspiration.

The averaged index (Table 1) would be plausible to farmers for cases (1) and (2), requiring some extension work to make them aware of basic crop physiology. Case (3), however, suggests that the averaged index is still too imprecise to capture spatial variation of factors that determine yield at some sites.

Farmers indicated crop durations ranging 60–90 days, depending on the microclimate of their sites because crop duration is largely under thermal control. The averaged insurance index covers a period of 90 days [16], [17], [18], but needs to be modified for shorter-duration crops. Further analysis of the farmers’ curves of water needs curves shows higher demands on soils other than *tierras negras*, which is in line with Díaz Nieto et al.’s recommendation to tailor indices to different soils [16], [17].

### 2. Representation of Spatially Varying Drought Risk

Farmers’ perceived risk of drought coincided with their perception of spatial variability of climate. Risk of drought was highest for the drier, warmer, and lower altitude sites. A single drought causes the highest damage in the moderate zones where drought is less likely but where asset exposure is high. This perception pattern of drought risk fits well to the analytical concept of risk as the product of damage potential and probability of an adverse event.

In general, farmers’ perceptions of drought risk coincided with the probabilities derived from the simulations, although farmers tended to underestimate the spatial differences compared with simulated values.

Farmers’ perceptions of climate were strongly dependent on the scale at which they operate, often discriminating as little as 1–2 km, and confirming the need for spatial resolution of 1 km to keep spatial basis risk low. For example, in San Dionisio the probability of a rainfall deficit of 60 mm at Wibuse is 0.15 but 10 km away at Las Cuchillas it is 0.66, which farmers knew had a higher risk of drought.

### 3. Recommendations for Further Methodology Improvement

We confirm that data of farmers’ perceptions and preferences support Díaz Nieto et al.’s methodology [16], [17], [18]. Technically, spatial basis risk can be lowered through higher resolution analysis, which from the viewpoint of the insured offers contracts tailored to their microclimate and soils.

#### 3.1 Modeling of indices

Díaz Nieto et al.’s averaged rainfall index [16], [17], [18] was based on MarkSim’s built-in climate surface with 10 arc-minute resolution. WorldClim’s surface with 30 arc-second resolution permits the method to take account for the research area’s heterogeneity. Moreover, the DSSAT crop simulations from which rainfall indices are derived need to take account of:

1. Adjust the sowing window (15 April–15 May) to those actually used by the target farmers. Díaz Nieto identified sowing window as critically important as it has a big influence on the periodical weighting of precipitation in the index [53] (cf. Table 1).
2. Soil characteristics are very important and the simulations should use data for the actual soils in the target area, including water characteristics, mineral composition, organic matter, and chemical data for each layer. Simulations should ideally include a range of terrain slopes and corresponding adjustments to the infiltration characteristics. Where possible the simulations should include comparison with field data to ensure satisfactory calibration. It should be tested whether offering two insurance instruments for soils of contrasting texture captures farmers’ distinction between *tierras negras* and *tierras blancas* and in doing so adequately reduces basis risk compared with one instrument for an average soil.
3. Farmers should be able to opt for indices for crop durations of 60, 75 and 85 days, depending on their sites’ microclimates (cf. Table 2). It is therefore adequate to compute indices for each of the climatic zones (*fresca*, *cálida*, *caliente*) as perceived by farmers.
4. Farmers reported that the duration of the *postrera* crop is about 10 days shorter than the *primera*. We need to know if this is a temperature effect captured by DSSAT and whether the *postrera* needs different weighting patterns, i.e. another set of indices.
5. Farmers often mentioned the specific microclimate on their fields that influenced cultivar perfomance. DSSAT can take account of slope and aspect, which, if there was perceived to be a demand, could readily be simulated to provide the basis for an appropriate insurance product. Still, the feasibility of offering a multitude of insurance products would depend on the demand and the willingness of the insurer to meet it. Technically there is no problem.
6. Farmers did not perceive differences in water demands between *mejoradas* and *criollas* cultivars. If there are differences, their genetic coefficients need to be determined to permit DSSAT to simulate them satisfactorily.

#### 3.2 Calculating drought probabilities on which to base insurance premiums

MarkSim-generated deficit probabilities in 1 km spatial resolution exhibit consistent spatial patterns, which agree largely with farmers’ perceptions of spatial climate variation. However, some aspects still need work:

1. An insurer would be interested in basing calculations on validated probability values. MarkSim has been validated a number of times [18], [25], [54], [55], [56], [57], [58]. Local validation is desirable, but practically difficult because of the uncertain quality of the historical meteorological data.
2. Spatial basis risk faced by a farmer is lowered by simulating site-specific probabilities at 1-km resolution and making premiums more realistic. The important task is to install sufficient rain gauges to capture the trigger event for specific farmers. This would also bring more evidence about the reliability of simulated drought probabilities. In Kenya, the Syngenta Foundation installs automatic rain gauges on cellphone towers, whose density is sufficient to provide adequate cover.

#### 3.3 Technical aspects

1. Farmers often grow drybean concurrently at different sites with different deficit probabilities. GPS equipment can provide the coordinates of each site to select the appropriate product. Similarly a group of neighbouring farmers doing cooperative agriculture could be provided with a group policy.
2. Farmers synchronised sowing with adequate soil water so that sowing failures were not relevant and need not to be considered in the insurance. In contrast, failures due to heavy rain at sowing and crop damage at harvest seem important, but lay outside the scope of this study.
3. Farmers partially knew about the ENSO phenomenon and corresponding local climate effects. MarkSim captures the variability caused by the ENSO phenomenon within its historical weather data, but does not explicitly generate weather that takes account of it. This is a clear area where refinement of MarkSim would be useful, and where adverse selection could be a problem.
4. For maximum transparency, it is necessary that farmers understand how the index is calculated and how premiums and indemnities are determined, which requires the involvement of the agricultural extension services.

### Conclusions

The study contributes to expanding rural households’ risk management strategies via formal insurance. The study’s approach emphasizes that perceptions and preferences of potential customers must be included in the process of developing acceptable insurance products.

Farmers’ perceptions supported the expectation that spatial basis risk merits special attention in mountainous areas, which has consequences for the design of appropriate indices. Firstly, the insurance has to specify what it undertakes to cover. To make basis risk transparent to farmers, they should be allowed to choose between a reasonable number of standardized contracts that take account of microclimate and soils and offer variable indices that give different levels of coverage, and of course, premiums. In the end it is the individual farmer who will decide if basis risk, and hence the insurance product, is acceptable or not.
